# Effects of Weather, Time, and Pollution Level on the Amount of Particulate Matter Deposited on Leaves of *Ligustrum lucidum*

**DOI:** 10.1155/2015/935942

**Published:** 2015-01-01

**Authors:** Huixia Wang, Hui Shi, Yanhui Wang

**Affiliations:** ^1^Research Institute of Forest Ecology, Environment and Protection, Chinese Academy of Forestry, Beijing 100091, China; ^2^School of Environmental and Municipal Engineering, Xi'an University of Architecture and Technology, Xi'an 710055, China

## Abstract

This paper investigated the spatial and temporal variations in the amounts of PM accumulated on leaves of *Ligustrum lucidum*, a common evergreen tree species in North China. The effects of rainfall and wind on the amounts of PM deposited on foliage were also determined. The amounts of PM (g·m^−2^) retained by leaves of *L. lucidum* differed significantly among the sites (from 0.96 to 5.56) and over time (from 2.51 to 4.48). The largest amounts of PM on foliage of *L. lucidum* were observed on plants growing at the most polluted site. During the year, the highest and lowest accumulation of PM occurred in November and August, respectively. A considerable proportion of the accumulated PM on leaves was removed by rainfall events (28–48% of PM) and strong winds (27–36% of PM), and more precipitation or higher maximum wind speed could remove more PM from leaves. Rainfall removed mainly large and coarse particles, while fine particles adhered more strongly to the foliage. These results suggested that the effects of local weather conditions (e.g., rainfall, strong wind), different seasons, and pollution levels should be considered in evaluating total PM accumulation on leaves.

## 1. Introduction

Rapid urbanization, industrialization, rural-urban migration, and the growing number and use of vehicles in recent years in cities have contributed to high concentrations of air pollutants, with particulate matter (PM) pollution being one of the most serious environmental problems [1, 2]. Atmospheric PM is mainly of anthropogenic origin (e.g., road traffic, industrial activity, domestic heating, and construction activity) and comprises a mixture of heavy metals, black carbon, polycyclic aromatic hydrocarbons, and other substances [2]. Current regulatory and research initiatives involving PM are driven by its effects on human health (e.g., cancer, heart disease, cardiovascular disease, eye irritation, respiratory disease, and asthma) [3], on visibility, and on the function of managed and natural ecosystems [4]. Therefore, reducing the PM concentration in ambient air is considered to be one of the most important protection tasks at present.

Reducing the sources of PM can improve urban air quality and lower the threat to human health, but phytoremediation is believed to be an additional and helpful measure that can be used to alleviate the air pollution by filtering and absorbing some PM through forest crown/leaves [1, 2, 5–7]. In USA, urban vegetation could remove about 21.49 × 10^4^ t of PM annually [8]. In the Greater London Authority, urban trees with a cover of 20% were estimated to remove 852–2121 t of PM10 (particulate matter with aerodynamic diameter <10 *μ*m) annually, representing 0.7–1.4% of PM10 coming from the urban boundary layer [9]. In urban Guangzhou, the PM retention amount of 18 species ranged between 0.066 and 1.831 g*·*m^−2^ [10]; the leaves of* Ficus virens*,* Ficus microcarpa*,* Bauhinia blakeana*, and* Mangifera indica* could remove 8013 t of PM per year [11]. These studies provide valuable information on PM retention effects by plants. However, in most of these studies, PM accumulation was analyzed only at one time during or at the end of the growing season or using the simulation method [2, 5–7, 9–11]. Because of changes in the characteristics of vegetation over time, and in local environmental and meteorological conditions, there are also large variations in atmospheric PM removal by leaves [12–14]. Weather conditions, especially rain [15–17] and wind, are probably the main drivers of the variations of PM accumulation on leaves over time. After deposition, rain may remove some of the PM from leaf surfaces [15–17]. It is important to analyze the wash-off or blow-off effects of precipitation or wind, respectively, to estimate accurately the amount of PM retained on leaves during a season or a year. However, to our knowledge, few studies have examined the variations of PM accumulation by leaves with the studies of Przybysz et al. [15], Rodríguez-Germade et al. [16], and Wang et al. [17], which focused on the wash-off effect of precipitation, being notable exception. This study attempts to address this knowledge gap.

This study was carried out in urban Xi'an, which is in North China, an area with temperate monsoon climate, and experienced a tremendous development.* Ligustrum lucidum*, a common evergreen tree species, is selected as the test material because of its prevalence in urban areas in the study area. We used this species as the test material to observe variations in PM deposited on leaves over a whole year, at different urban environments, and to examine the effects of rain and wind on removal of PM from the leaves.

## 2. Materials and Methods

### 2.1. Study Area

**Figure 1:**
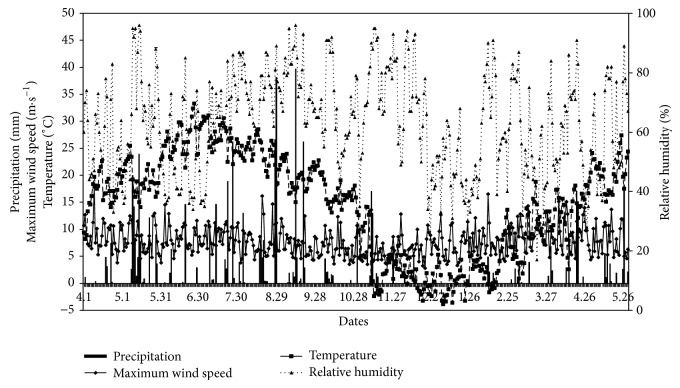
Diurnal variations of precipitation, maximum wind speed, and mean relative humidity.

Xi'an is a historic city and one of the most famous tourism cities in China. The city has more than eight million people and thus a sizeable volume of traffic exists. This area has a temperate monsoon climate. The southern district of the city, a rapid development zone in recent years, was selected as the sampling area. Climatic data recorded by the National Reference Climatological Station (Jinghe) giving daily values during the study period (Figure 1) were downloaded from China Meteorological Data Sharing Service System (http://cdc.cma.gov.cn/) [18]. During the study period, the daily precipitation ranged from 0.1 mm to 39.7 mm, the maximum wind speed from 2.8 m*·*s^−1^ to 17.8 m*·*s^−1^, the daily mean temperature from −3.9°C to 33.3°C, and the daily mean relative humidity from 17% to 96% (Figure 1).

### 2.2. Plant Material

An evergreen tree species of* L. lucidum* (Oleaceae) was selected as the test material. The shape of the leaves was ovate to ovate-lanceolate. The leaves have a smooth cuticle, no trichomes, and the shape of epicuticular wax was membrane.

### 2.3. Effects of Pollution Level on PM Retained by Leaves

**Figure 2:**
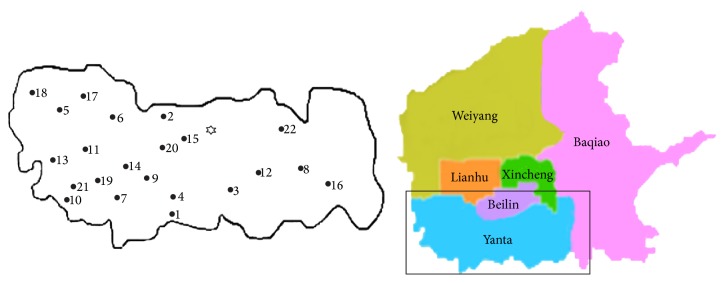
Study area (black circles indicated the sampling sites with different pollution level; asterisk indicated the sampling site of Xi'an University of Architecture and Technology).

Twenty-two sampling sites (Figure 2) were selected to investigate the effects of pollution level on leaf PM accumulation (sampling 1). Two of these sites were located in factories, where the plants grew near the door of the factory. Two of these sites were located in residential area, where the plants grew in the center of the community. Two of these sites were green lands, and distance between plants and road exceeded 200 m. The other sixteen sites were busy highways, main roads, and submain roads, where the plants grew on the roadside. Some studies have shown that the pollution level was directly proportional to traffic density [14, 19] and different land-use classes that were associated with different human activities [20]; thus, the traffic density combining the surrounding environmental conditions was used to indicate the pollution level in this study. The traffic density at each site was monitored at 7:00–9:00, 11:30–14:00, and 17:00–19:00 on each sampling day. The traffic density and the characteristics of the environment surrounding the sampling sites are shown in Table 1.

### 2.4. Effects of Time and Weather on PM Retained by Leaves

The campus of Xi'an University of Architecture and Technology was selected to investigate the effects of time (sampling 2) and weather (sampling 3) on the amounts of PM accumulated on leaves. Since the campus is located in the center part of the city and adjacent to Yanta Road (a busy road of the city), the plants were mainly exposed to urban road dust, motor vehicle exhaust, and natural dust sources. The distance between plants and road was nearly 50 m.

### 2.5. Leaf Sampling

For sampling 1, the leaves of* L. lucidum* were collected from May 1 to May 7, 2010. For sampling 2, the leaves were collected monthly from April 2009 to March 2010. For sampling 3, the leaves were collected under certain weather conditions from March 21 to May 3, 2010, as follows: after 6 and 12 continuously clear days, after rainfall events with 10.4 and 31.9 mm precipitation, and in windy conditions (with maximum wind speeds of 11.1, 7.1, 12.1, 15.2, and 4.4 m*·*s^−1^).

Samples were collected from five plants at each sampling site and on any given sampling day. All sample trees were in the same age and were suffering from no obvious pests or disease. Fully expanded and turgid leaves which sprouted at the same time were collected. They were collected from the inner and outer canopy of N, W, S, and E facing aspects at a height of approximately 2–5 m above ground level with a pruner. A sample of 30–50 pieces of leaves was collected from each tree and then bulked to give a total of 150–250 pieces of leaves per sampling site and sampling day. All sample leaves were kept in a cool box both during transport and in the laboratory until analysis. It should be noted that leaf samples for sampling 2 and sampling 3 were collected from the same trees on any given sampling day.

### 2.6. Quantitative Assessment of PM Retained on Leaves

The amount of PM retained on leaves was examined according to Prusty et al. [14]. Each sample had three repetitions, and each repetition consisted of 15–20 pieces of leaves. First, the plant material was immersed in 250 mL distilled water. Next, each leaf was held individually with tweezers, and the PM deposited on leaf surfaces was washed using a no-hair-loss brush. And then, the leaves were washed using about 5 mL distilled water. The entire procedure typically took less than 10 min, minimizing the water-soluble particles. The total hemisurface (i.e., one side) leaf area (*A*, m^2^) was measured using Image J software (Version 1.46; National Institutes of Health, Bethesda, MD, USA) after scanning (HP Scanjet G2410, HP, Japan). For the filtration procedure, we used a filter membrane with a pore size of 0.45 *μ*m (Hai Cheng Shi Jie Filtering Equipment Ltd. Co., Beijing, China). The filter membranes were preweighed (*W*_1_, g) after 24 h drying at 40°C using a balance with 0.1 mg accuracy (FA2004; Shanghai Precision Instruments, Shanghai, China) which located in a balance room. Before filtration, the washing solution was hand-shaken for several seconds to resuspend all washed particles. The filtration was carried using a 47 mm glass filter funnel with stopper support assembly (Millipore Corp., Bedford, MA, USA) connected to a vacuum pump (SHB-III; Greatwall Scientific Industrial and Trade, Co., Ltd., Zhengzhou, China). Water-soluble particles and the particles with a diameter of <0.45 *μ*m remaining in the solution were not considered here. Loaded filter membranes (*W*_2_, g) were subsequently dried at 60°C in an oven to a constant weight, stabilized in the balance room for 30 min, and then reweighed. The amount of PM retained on leaves (*W*, g*·*m^−2^) was calculated as follows: *W* = (*W*_2_ − *W*_1_)/*A*.

### 2.7. Scanning Electron Microscopy (SEM)

A scanning electron microscope (JSM-6510LV, JEOL, Japan) was used to obtain the characteristics of the adaxial and abaxial surfaces of* L*.* lucidum* before and after a rainfall event (31.9 mm). Three leaves before and after rainfall event were selected, respectively. For each leaf, approximately 2 × 0.5 cm^2^ was cut with a razor blade from the middle part of the leaf discarding the mid vein. Next, these samples were mounted on aluminum stubs with double-sided adhesive tape, with adaxial and abaxial surfaces exposed side by side on the same stub. Then the specimens were examined and photographed under an accelerating voltage of 10 kV, a working distance of 10–13 mm, and magnifications of 500x, 1000x, and 2000x. For each magnification, each leaf, and each leaf side, five randomly chosen fields were observed. A multiscale analysis at magnifications of 500x, 1000x, and 2000x was carried out to cover different fields of view in order to see the large to small particles on the leaf surface in the SEM [21]. From the 500x dimension of the initial field of view we zoomed in to 1000x and then 2000x. The 500x image (260 *μ*m × 196 *μ*m) was examined first; the 1000x (130 *μ*m × 98 *μ*m) and 2000x (65 *μ*m × 49 *μ*m) images were within the same field of view. In this way, we obtained a total of 90 images of leaves on each given sampling day.

### 2.8. Analysis of Particle Size Distribution

The particle size distributions before and after rainfall event were examined and analyzed through 10 pieces of micrograph at each magnification (500x, 1000x, and 2000x). On each micrograph, 20 particles were randomly selected for measuring the length and width using Image J software. The average of the length and width of the particle was considered as the diameter of the measured particle. In this way, the diameter of a total of 1200 particles (20 (particle number per piece of micrograph) × 10 (pieces of micrographs) × 3 (magnification) × 2 (leaf side)) per leaf was obtained.

### 2.9. Statistical Analysis

Statistics analysis was performed with SPSS 19.0 software (IBM, USA). Analyses of variance (ANOVA) were applied to determine if significant differences in the amount of leaf-deposited PM occurred among different sampling sites and sampling time. When analysis of variance indicated significant differences among sampling sites and sampling time, Tukey's honestly significant difference (Tukey's HSD) tests were performed to determine which exhibited significant differences. The differences in the amount of PM among different weather conditions were also used by ANOVA and Tukey's HSD. A given effect was assumed to be significant at *P* < 0.05.

## 3. Results

### 3.1. Leaf PM Retention Amounts at Different Sampling Sites

**Figure 3:**
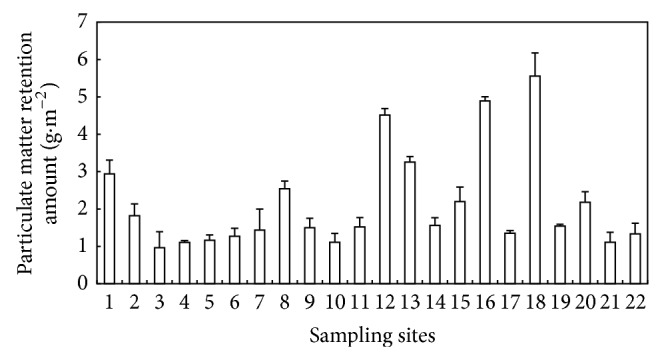
The amount (g·m^−2^) of PM deposited on leaves of* L*.* lucidum* grown at 22 sampling sites differing in pollution level (data are mean ± SD).

The PM retention amounts on leaves of* L*.* lucidum* showed significant difference among sampling sites (Figure 3, *P* < 0.001). Among all of the investigated sites, the amount of PM accumulated on leaves ranged from 0.96 g*·*m^−2^ (sampling site 3, an urban green area) to 5.56 g*·*m^−2^ (sampling site 18, an industrial area).

### 3.2. In-Season Variation of Leaf PM Retention Amounts

**Figure 4:**
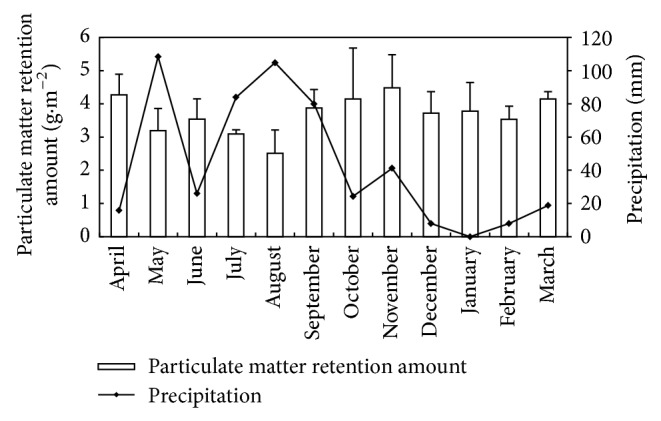
Comparison of the temporal variations of monthly rainfall totals (mm) and mean leaf accumulation PM amount (g·m^−2^, data are mean ± SD) by leaves of* L*.* lucidum*.

There were clear differences in PM accumulation on leaves of* L*.* lucidum* during the whole year (Figure 4, *P* < 0.001). The amount of PM retained on leaves was highest in November (4.48 g*·*m^−2^) and lowest in August (2.51 g*·*m^−2^). In April, October, November, and March, the amount of PM accumulated on leaves exceeded 4 g*·*m^−2^, while it ranged from 3 to 4 g*·*m^−2^ in the other months, except for August (Figure 4).

### 3.3. Effects of Rainfall and Wind on Leaf PM Retention Amounts

**Figure 5:**
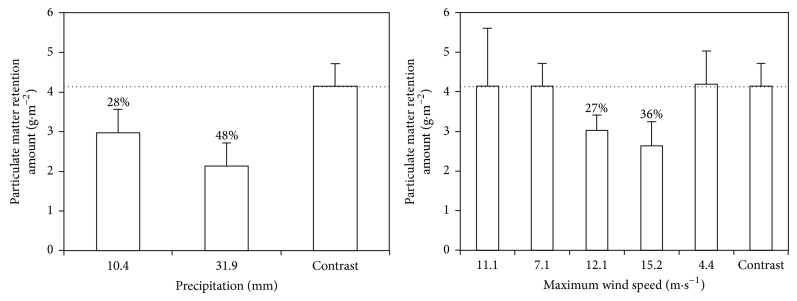
The effects of precipitation (mm) and maximum wind speed (m·s^−1^) on leaf PM retention amounts (g·m^−2^, data are mean ± SD).

Rainfall events removed large quantities of PM from leaves of* L*.* lucidum*. In total, 28% and 48% of accumulated PM were washed off from leaves with 10.4 mm and 31.9 mm precipitation, respectively (Figure 5(a)). Wind at speeds lower than 11.1 m*·*s^−1^ could not blow off the PM deposited on leaves. Stronger winds of 12.1 m*·*s^−1^ and 15.2 m*·*s^−1^ blew off 27% and 36%, respectively, of the accumulated PM from leaves (Figure 5(b)).

### 3.4. Variations in Particles Size before and after Rainfall Event

**Figure 6:**
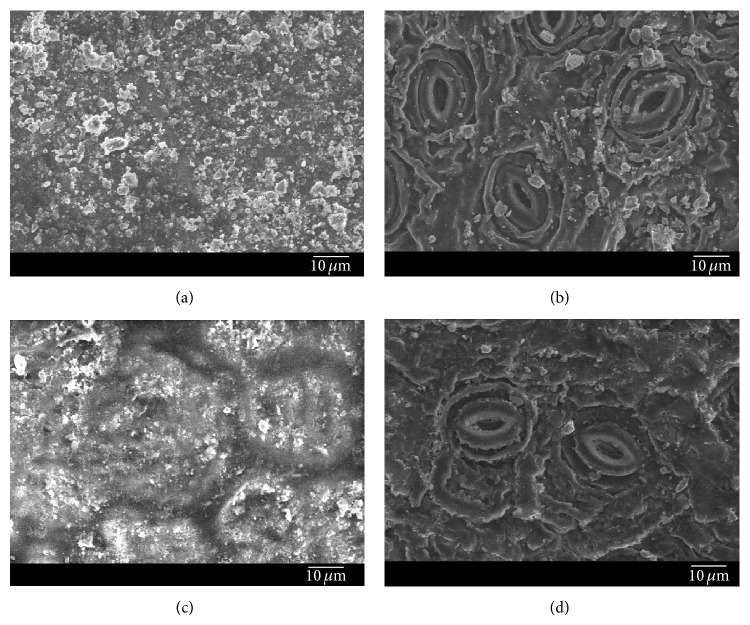
The upper ((a), (c)) and lower ((b), (d)) surfaces of leaves of* L. lucidum *before ((a), (b)) and after ((c), (d)) a rainfall event.

**Figure 7:**
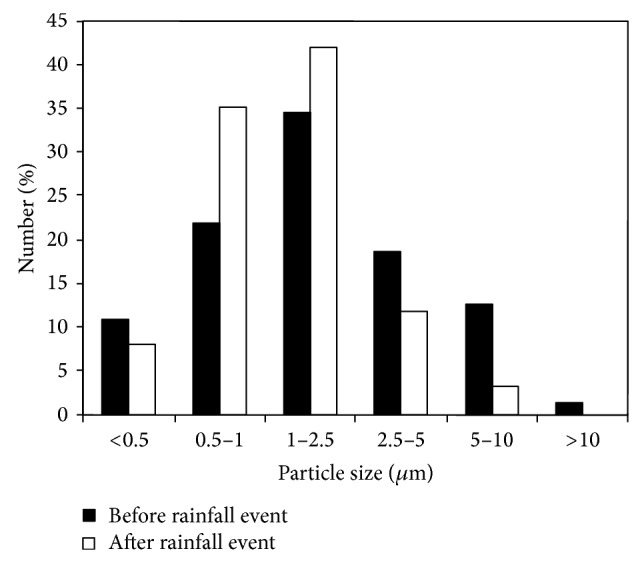
The particle size distribution before and after a rainfall event.

Analyses of PM deposited on leaves before and after a rainfall event of 31.9 mm (Figures 6 and 7) showed that the proportion of large particle (PM with diameter >10 *μ*m) decreased from 1.4% to 0%. The particles with diameters of 5–10 *μ*m and 2.5–5 *μ*m decreased from 12.6%, 18.6% to 3.4%, 11.6%, respectively, while the proportion of fine fraction (PM with diameter <2.5 *μ*m) increased.

## 4. Discussion

### 4.1. Differences in PM Retention among Locations

The greater amount of PM was found on the foliage of plants grown at the more polluted site. The concentration of PM in the air has been identified as an important factor in foliar PM deposition [2, 6, 9, 14, 15]. Przybysz et al. [15] found that three evergreen species,* Taxus baccata*,* Pinus sylvestris*, and* Hedera helix*, accumulated more PM on leaves at a heavily polluted site than at less polluted sampling sites. Sæbø et al. [2] reported that, for the same species at two locations, the amount of leaf-deposited PM was higher in Poland than in Norway, consistent with the higher air pollution in Poland than in Norway. In a study of foliar PM retention near the national highway at Sambalpur, Orissa, India, the amount of leaf-deposited PM increased with increasing number of vehicles [14].

The microenvironmental and microclimatic conditions are generators of individual variation and directly influence leaf traits, such as stomatal characteristics [22–25]. Stomatal density significantly increased from suburban green, urban green, urban area, and industrial area [26]. A large stomatal density may result in increased transpiration; this can make particles more deliquescent and can increase deposition rates [27]. Transpiration of water through stomata also can cool leaf surfaces and thus increase PM deposition by thermophoresis [28]. These may also partly explain the higher ability of leaves to capture PM under more polluted conditions when compared with comparatively clean areas.

### 4.2. In-Season Variations of Leaf PM Retention Amounts

In this study, we observed the variations in PM retention on leaves of* L*.* lucidum* throughout the growing season. Higher values were obtained in months with lower precipitation (e.g., October, November), and lower values were obtained in the month with the higher precipitation (August), as reported in other studies [14–16, 29]. This may be related not only to the different frequency and intensity of rainfall events, but also to the inconsistent PM emissions into the atmosphere during the experiment period. Prajapati and Tripathi [30] found that less PM accumulated during the rainy season compared with winter and summer, which they attributed to the washing effect of precipitation. The greater amounts of deposited PM in winter could be attributed to the wet surfaces of leaves during lighter rainfall events which may help to capture PM, and gentle breezes and foggy conditions prevent PM dispersion [30]. Meanwhile, ambient PM concentrations in winter were always higher than those in summer and rainy days [31]. The higher PM concentrations in the atmosphere in winter could lead to higher deposition rates and greater amounts of PM being retained on leaves, compared with those reported in summer.

### 4.3. Effects of Rainfall and Wind on Leaf PM Retention Amounts

In nature, PM deposited on leaf surfaces can be washed off by rain or blown off by wind [15–17]. A comparison of leaves collected under certain environmental conditions showed that rainfall events and strong winds removed a considerable proportion of PM deposited on leaves of* L. lucidum* during the season. The more precipitation or higher maximum wind speed could remove more PM from leaves. Raindrops or wind had high kinetic energy that they might remove many particles by splashing/impacting with the leaf surfaces [13]. In the study of Przybysz et al. [15], which was conducted in Stavanger, Norway, 30–41% of PM was washed off from* P. sylvestris* by simulated rainfall (20 mm). The fraction of large particles made up the greatest mass proportion (33–42%) of removed PM, followed by the coarse fraction (25–36%), and then the fine fraction (21–30%). In a study conducted in Madrid, Spain, Rodríguez-Germade et al. [16] demonstrated that rainfall washed off a portion of PM that deposited on the leaves of* Platanus hispanica*. In another study, approximately 50% of PM was washed off by rainfall (14.5 mm) [17]. However, Freer-Smith et al. [32] found that the amounts of coarse and fine PM on foliage before and after precipitation were not significantly different; this finding suggested that once deposited, PM is not easily washed off from leaf surfaces. Another study reported that simulated rainfall could not wash off PM with a diameter smaller than 5 *μ*m and especially that with a diameter smaller than 1 *μ*m, from leaves of* Euonymus japonicus* [33]. To analyze accurately PM accumulation, resuspension of PM by wind should also be considered. Ould-Dada and Baghini [34] found that a small proportion of PM could be resuspended by wind at a speed of 5 m*·*s^−1^. In a study conducted in Beijing, China, the amount of leaf-deposited PM was not significantly different before and after a strong wind event (<10.4 m*·*s^−1^) [17]. Furthermore, the number of PM removed from leaves related closely with particle diameter, and large and coarse particles were more easily washed off or blown off than the fine particles. The deposition of PM on leaves depends not only on leaf surface microstructure but also on PM diameter. The fine particle always deposited in the microstructure (e.g., hollows, grooves) [35] that is not easily removed.

## 5. Conclusions and Suggestion

It can be shown in this study that the amount of PM retained by leaves of* L*.* lucidum* differed significantly among sites and over time.* L*.* lucidum*, accumulating the largest amounts of PM, were planted at the most polluted site. The highest PM accumulation occurred in November, the month with the lowest precipitation, while the lowest was in August, the month with the highest precipitation. Rainfall events and strong winds affected the amount of PM accumulated on leaves. In total, 28% to 48% of PM on leaves was washed off by rain, and 27% to 36% was blown off by wind. Rainfall removed mainly large and coarse particle, while fine particles adhered more strongly to the foliage.

These results suggest that it is important to understand the spatial and temporal variations of accumulation, wash-off, and blow-off of accumulated PM to determine the effects of vegetation on local air quality. The results of the present study indicated that analyzing leaf-deposited PM at one time point during or at the end of the season only partly explains the ability of leaves to capture PM from the air. To better understand PM accumulation on leaves, different pollution levels, different seasons, precipitation, and wind speed should also be considered when evaluating the total PM removal capacity of plants. However, there is still much to learn about the potential of plants to accumulate PM, as there is still a lack of robust data on the effects of rain and wind on leaf PM accumulation. These topics should be investigated in further research.

## Figures and Tables

**Table 1:** The measured traffic density and environmental conditions in areas around sampling sites (— indicates not measured).

Number	Traffic density/(h^−1^)	Surrounding environmental conditions
1	5170 ± 1104	Tremendous traffic density.
2	3252 ± 170	Heavy traffic density.
3	1114 ± 172	Tang Dynasty Lotus Garden, large flow of people.
4	1936 ± 265	Located in the south of Chang'an South Road, the west of Yanzhan Road, well greening environment around.
5	—	Located in Tangxing Road, good environment condition in the garden.
6	604 ± 15	Located in the cultural and educational area, small traffic density.
7	2358 ± 182	East of Northwest Water Power Building, no obvious pollution sources.
8	1688 ± 397	West of Xi'an University of Technology (Qujiang campus), east of the Science Park of Xi'an Jiaotong University, surrounded by ceramic factory, building materials warehouse.
9	764 ± 108	East of Cheng Nan Coach Station, mainly affected by auto exhaust.
10	1888 ± 334	Well greening environment around, no obvious pollution sources.
11	1442 ± 228	Adjacent to Mu Pagoda Park, well greening environment around.
12	2062 ± 649	Many buildings under construction, a lot of lime deposited on leaf surfaces, and heavy traffic density.
13	—	Next to special steel factory.
14	1612 ± 129	Located in the south of Hanguang Road, well greening environment around, no obvious pollution sources.
15	2972 ± 567	Adjacent to second ring of Southern Xi'an, affected by vehicles from second ring of southern Xi'an and Yanta North Road.
16	538 ± 70	Small traffic density, a lot of garbage piling up around, existence of the case of waste incineration.
17	2532 ± 333	Heavy traffic density, no obvious pollution sources.
18	—	Affected by chemical factory.
19	504 ± 93	Residential area, north of Xi'an Lian Chuang Electronic Group, a small range of constructions in the west.
20	1794 ± 263	Commercial district, large flow of people.
21	1556 ± 281	Medium traffic density, adjacent to wholesale base of wood, steel, and building materials.
22	1920 ± 336	Adjacent to Xi'an C. P. Pharmaceutical Co., LTD., medium traffic density.

## References

[1] Kardel F., Wuyts K., Maher B. A., Hansard R., Samson R. (2011). Leaf saturation isothermal remanent magnetization (SIRM) as a proxy for particulate matter monitoring: inter-species differences and in-season variation. *Atmospheric Environment*.

[2] Sæbø A., Popek R., Nawrot B., Hanslin H. M., Gawronska H., Gawronski S. W. (2012). Plant species differences in particulate matter accumulation on leaf surfaces. *Science of the Total Environment*.

[3] Pope C. A., Burnett R. T., Thurston G. D. (2004). Cardiovascular mortality and long-term exposure to particulate air pollution: epidemiological evidence of general pathophysiological pathways of disease. *Circulation*.

[4] Grantz D. A., Garner J. H. B., Johnson D. W. (2003). Ecological effects of particulate matter. *Environment International*.

[5] Escobedo F. J., Kroeger T., Wagner J. E. (2011). Urban forests and pollution mitigation: analyzing ecosystem services and disservices. *Environmental Pollution*.

[6] Nowak D. J., Hirabayashi S., Bodine A., Hoehn R. (2013). Modeled PM_2.5_ removal by trees in ten U.S. cities and associated health effects. *Environmental Pollution*.

[7] Popek R., Gawrońska H., Wrochna M., Gawroński S. W., Sæbø A. (2013). Particulate matter on foliage of 13 woody species: deposition on surfaces and phytostabilisation in waxes—a 3-year study. *International Journal of Phytoremediation*.

[8] Nowak D. J., Crane D. E., Stevens J. C. (2006). Air pollution removal by urban trees and shrubs in the United States. *Urban Forestry and Urban Greening*.

[9] Tallis M., Taylor G., Sinnett D., Freer-Smith P. (2011). Estimating the removal of atmospheric particulate pollution by the urban tree canopy of London, under current and future environments. *Landscape and Urban Planning*.

[10] Liu L., Guan D., Chen Y. D. (2013). Morphological structure of leaves and dust-retaining capability of common street trees in Guangzhou Municipality. *Acta Ecologica Sinica*.

[11] Liu L., Guan D., Peart M. R., Wang G., Zhang H., Li Z. (2013). The dust retention capacities of urban vegetation-a case study of Guangzhou, South China. *Environmental Science and Pollution Research*.

[12] Wang H., Shi H., Li Y., Yu Y., Zhang J. (2013). Seasonal variations in leaf capturing of particulate matter, surface wettability and micromorphology in urban tree species. *Frontiers of Environmental Science and Technology*.

[13] Neinhuis C., Barthlott W. (1998). Seasonal changes of leaf surface contamination in beech, oak, and ginkgo in relation to leaf micromorphology and wettability. *New Phytologist*.

[14] Prusty B. A. K., Mishra P. C., Azeez P. A. (2005). Dust accumulation and leaf pigment content in vegetation near the national highway at Sambalpur, Orissa, India. *Ecotoxicology and Environmental Safety*.

[15] Przybysz A., Sæbø A., Hanslin H. M., Gawroński S. W. (2014). Accumulation of particulate matter and trace elements on vegetation as affected by pollution level, rainfall and the passage of time. *Science of the Total Environment*.

[16] Rodríguez-Germade I., Mohamed K. J., Rey D., Rubio B., García Á. (2014). The influence of weather and climate on the reliability of magnetic properties of tree leaves as proxies for air pollution monitoring. *Science of the Total Environment*.

[17] Wang L., Hasi E., Liu L., Gao S. (2006). Effects of weather condition in spring on particulates density on conifers leaves in Beijing. *Chinese Journal of Ecology*.

[18] China Meteorological Data Sharing Service System http://cdc.cma.gov.cn.

[19] Pal A., Kulshreshtha K., Ahmad K. J., Behl H. M. (2002). Do leaf surface characters play a role in plant resistance to Auto-exhaust pollution?. *Flora*.

[20] Shao T. J., Zhao J. B., Ma L. (2008). The spatial temporal variation characteristics of air pollutants in X'ian. *Journal of Arid Land Resources and Environment*.

[21] Sternberg T., Viles H., Cathersides A., Edwards M. (2010). Dust particulate absorption by ivy (*Hedera helix* L) on historic walls in urban environments. *Science of the Total Environment*.

[22] Alves E. S., Moura B. B., Domingos M. (2008). Structural analysis of *Tillandsia usneoides* L. exposed to air pollutants in São Paulo City-Brazil. *Water, Air, and Soil Pollution*.

[23] Gray J. E., Holroyd G. H., van der Lee F. M. (2000). The HIC signalling pathway links CO_2_ perception to stomatal development. *Nature*.

[24] Liu-Gitz L., Britz S. J., Wergin W. P. (2000). Blue light inhibits stomatal development in soybean isolines containing kaempferol-3-*O*-2^G^-glycosyl-gentiobioside (K9), A unique flavonoid glycoside. *Plant, Cell & Environmen*.

[25] Wali B., Iqbal M. (2007). Anatomical and functional responses of *Calendula officinalis* L. to SO_2_ stress as observed at different stages of plant development. *Flora: Morphology, Distribution, Functional Ecology of Plants*.

[26] Kardel F., Wuyts K., Babanezhad M. (2010). Assessing urban habitat quality based on specific leaf area and stomatal characteristics of *Plantago lanceolata* L. *Environmental Pollution*.

[27] Burkhardt J., Koch K., Kaiser H. (2001). Deliquescence of deposited atmospheric particles on leaf surfaces. *Water, Air, and Soil Pollution*.

[28] Räsänen J. V., Holopainen T., Joutsensaari J. (2013). Effects of species-specific leaf characteristics and reduced water availability on fine particle capture efficiency of trees. *Environmental Pollution*.

[29] Matzka J., Maher B. A. (1999). Magnetic biomonitoring of roadside tree leaves: identification of spatial and temporal variations in vehicle-derived particulates. *Atmospheric Environment*.

[30] Prajapati S. K., Tripathi B. D. (2008). Seasonal variation of leaf dust accumulation and pigment content in plant species exposed to urban particulates pollution. *Journal of Environmental Quality*.

[31] Xi'an Environmental Protection Bureau Air quality daily [EB/OL]. http://www.xaepb.gov.cn/ajax/comm/pm25/newMapindex.jsp.

[32] Freer-Smith P. H., Beckett K. P., Taylor G. (2005). Deposition velocities to *Sorbus aria*, *Acer campestre*, *Populus deltoides* × *trichocarpa* “Beaupré”, *Pinus nigra* and × *Cupressocyparis leylandii* for coarse, fine and ultra-fine particles in the urban environment. *Environmental Pollution*.

[33] Wang Z. H., Li J. B. (2006). Capacity of dust uptake by leaf surface of *Euonymus japonicus* Thunb. and the morphology of captured particle in air polluted city. *Ecological Environment*.

[34] Ould-Dada Z., Baghini N. M. (2001). Resuspension of small particles from tree surfaces. *Atmospheric Environment*.

[35] Shi H., Wang H., Li Y., Liu X. (2011). Leaf surface microstructure of *Ligustrum lucidum* and *Viburnum odoratissimum* observed by atomic force microscopy (AFM). *Acta Ecologica Sinica*.

